# Enhancing Angioinvasion Assessment in Papillary Thyroid Cancer Via a Biomarker Panel Involving TAC, 8-OHdG, and Sortilin

**DOI:** 10.1210/clinem/dgae007

**Published:** 2024-01-05

**Authors:** Angelika Buczyńska, Maria Kościuszko, Iwona Sidorkiewicz, Aleksandra Anna Wiatr, Agnieszka Adamska, Katarzyna Siewko, Janusz Dzięcioł, Małgorzata Szelachowska, Adam Jacek Krętowski, Anna Popławska-Kita

**Affiliations:** Clinical Research Centre, Medical University of Bialystok, 15-276 Bialystok, Poland; Department of Endocrinology, Diabetology and Internal Medicine, Medical University of Bialystok, 15-276 Bialystok, Poland; Clinical Research Support Centre, Medical University of Bialystok, 15-276 Bialystok, Poland; Clinical Research Centre, Medical University of Bialystok, 15-276 Bialystok, Poland; Department of Endocrinology, Diabetology and Internal Medicine, Medical University of Bialystok, 15-276 Bialystok, Poland; Department of Endocrinology, Diabetology and Internal Medicine, Medical University of Bialystok, 15-276 Bialystok, Poland; Department of Human Anatomy, Medical University of Bialystok, 15-276 Bialystok, Poland; Department of Endocrinology, Diabetology and Internal Medicine, Medical University of Bialystok, 15-276 Bialystok, Poland; Clinical Research Centre, Medical University of Bialystok, 15-276 Bialystok, Poland; Department of Endocrinology, Diabetology and Internal Medicine, Medical University of Bialystok, 15-276 Bialystok, Poland; Department of Endocrinology, Diabetology and Internal Medicine, Medical University of Bialystok, 15-276 Bialystok, Poland

**Keywords:** papillary thyroid cancer, oxidative stress, angioinvasion, 8-OHdG, sortilin

## Abstract

**Context:**

Papillary thyroid cancer (PTC) aggressiveness and metastatic potential are closely associated with angioinvasion. Identifying angioinvasion accurately is imperative for treatment planning and prognosis.

**Objective:**

This study explores serum biomarkers, including 8-hydroxydeoxyguanosine (8-OHdG) and oxidative status markers (total oxidative capacity, total antioxidant capacity [TAC], and sortilin), as potential indicators of angioinvasion in PTC.

**Design:**

A cross-sectional study involving 50 angioinvasive patients with PTC (study group) and 30 patients with PTC with low-risk features (reference group). Serum levels of biomarkers were analyzed to determine their association with angioinvasion.

**Setting:**

Patients were recruited from Department of Endocrinology, Diabetology, and Internal Diseases, Medical University of Bialystok, Poland, ensuring representation from a diverse clinical context.

**Patients or Other Participants:**

Participants included patients with PTC, with 50 in the study group and 30 in the reference group. Selection criteria, matching characteristics, and participant completion rates were duly recorded.

**Intervention(s):**

Serum biomarkers were measured to evaluate their association with PTC angioinvasion.

**Main Outcome Measure(s):**

Primary outcome measures included serum levels of 8-OHdG, total oxidative capacity, TAC, and sortilin.

**Results:**

Serum levels of 8-OHdG and sortilin were significantly elevated in angioinvasive PTC, whereas TAC showed a notable decrease (all *P* < .01). A regression panel combining TAC, 8-OHdG, and sortilin demonstrated a high area under the curve value (0.963) for angioinvasion discernment.

**Conclusion:**

Measuring TAC, 8-OHdG, and sortilin levels may serve as potential biomarkers for identifying angioinvasion in PTC. The combined assessment of these biomarkers enhances angioinvasion discernment, aiding risk stratification and personalized treatment decisions. Further validation studies are required before integrating these biomarkers into routine clinical practice. The study adheres to the provided structure, providing concise and supported conclusions based on the results.

Papillary thyroid cancer (PTC) is the most prevalent type of thyroid malignancy, accounting for approximately 80% of all thyroid cancer cases (1). Although the overall prognosis for PTC is generally favorable, a subset of patients exhibits aggressive disease progression, leading to poorer outcomes (2). Angioinvasion, which involves the invasion of tumor cells into blood vessels, is a crucial factor associated with increased tumor aggressiveness and a higher risk of metastasis in PTC (3). Accurate identification of angioinvasion is essential for determining appropriate treatment strategies and predicting patient prognosis. However, current diagnostic methods, such as histopathological examination, suffer from subjectivity and interobserver variability, highlighting the need for reliable and objective biomarkers to aid the identification of angioinvasion in PTC (4).

Oxidative stress, defined by an imbalance between the production of reactive oxygen species (ROS) and the body's antioxidant defenses, has been associated with a poorer prognosis and angioinvasion in patients with PTC (5, 6). The oxidative stress is believed to expedite the progression of PTC by enhancing the proliferation and invasiveness of the cancer (7). Because thyroid tissue exhibits significant sensitivity to ROS, the hypothesis suggests that there might be a potential correlation between the level of ROS and prognosis or indicators of aggressiveness (6, 8, 9). Moreover, ROS have the capacity to inflict damage on cellular components, including DNA (10). One specific marker of DNA oxidation is 8-hydroxy-2'-deoxyguanosine (8-OHdG), which also reflects the extent of oxidative stress in cells (6, 11). Therefore, evaluating oxidative stress markers holds promise as a means of identifying angioinvasion in PTC (5, 12, 13). Elevated levels of 8-OHdG in patients with PTC with angioinvasion may indicate increased DNA damage caused by oxidative stress, potentially serving as a molecular marker for assessing the aggressiveness of the disease (14). Consequently, 8-OHdG has been proposed as a potential biomarker for identifying cases of aggressive PTC characterized by the presence of angioinvasion, wherein cancer cells infiltrate blood vessels. Additionally, sortilin, a protein intricately implicated in pivotal cellular mechanisms encompassing protein trafficking and signaling, has been linked to neoplastic proliferation, invasion, and metastasis across heterogeneous cancer categories (15). In the context of PTC, sortilin has been postulated to play a role in facilitating tumor invasion and metastasis. It is hypothesized to augment cancer cell migration and adhesion to blood vessels, thereby promoting angioinvasion and potential distant metastasis. Consequently, sortilin has emerged as a subject of interest as a potential marker for aggressive PTC cases. With the potential significance of oxidative stress and sortilin in thyroid cancer, there is a growing interest in exploring their utility as biomarkers for identifying angioinvasion in PTC.

The objective of this study is to evaluate the screening potential of newly identified markers for the detection of angioinvasion in patients diagnosed with PTC. Specifically, our investigation is centered on appraising the efficacy of quantifying oxidative stress markers, 8-OHdG, and sortilin levels as innovative indicators of PTC angioinvasion. By examining the screening utility of these markers, our research aims to contribute to the development of noninvasive and objective methods for identifying angioinvasion in patients with PTC. Ultimately, the findings from this study could enhance early detection, risk stratification, and personalized management of PTC, thereby improving patient outcomes and clinical decision-making.

## Material and Methods

This study was carried out at the Department of Endocrinology, Diabetology, and Internal Diseases, Medical University of Bialystok, Poland. The procedures were approved by the Local Ethics Committee of the Medical University of Bialystok, Poland, and written informed consent was obtained from each participant (R-I-002/491/2019).

### Studied Population

The patient inclusion criteria involved clinical laboratory tests and ultrasound imaging to diagnose all patients with PTC. The diagnoses were further confirmed through fine-needle aspiration biopsy and subsequent histopathological examination during thyroidectomy. For the purpose of this study, a total of 50 patients with angioinvasive PTC were included in the study group, whereas another 30 patients with PTC and very low risk were selected as the reference group (Table 1).

**Table 1.** Characteristics of patients with PTC

	Study group	Reference group
Number of patients	50	30
Median age (upper and lower quartiles)	54 (51.41; 64.22)	53 (50.11; 66.31)
Sex	M: 12	M: 8
	F: 38	F: 22
Menopausal status		
Premenopausal	11	13
Postmenopausal	27	9
Stage (TNM)	pT1a(m):11 pT1b: 15 pT1b(m): 6 pT2: 12 pT3/pT4: 6	pT1a:30
Patients diagnosed with angioinvasion	50	0
Patients without angioinvasion	0	30

Abbreviations: F, female; M, male; (m), multifocal; p, pathological; pT1a, tumor size ≤1 cm in greatest dimension limited to the thyroid; pT1b, tumor >1 cm but ≤2 cm in greatest dimension, limited to the thyroid; pT2, tumor size >2 cm but ≤4 cm, limited to the thyroid; pT3/pT4, tumor size >4 cm, with gross extrathyroidal extension; SE, standard error; TNM, Cancer tumor-node-metastasis classification (based on the characteristics of primary tumor site [pT]).

In the comprehensive study, we enrolled a total of 300 patients diagnosed with PTC. However, for the specific aims of this research, we chose to include only those individuals who exhibited clear evidence of angioinvasion. Our selection criteria were based on the fundamental principle of ensuring homogeneity within the study cohort. None of the patients was taking medications or had any other underlying conditions that could potentially influence peripheral oxidative stress or similar criteria. All patients were enrolled in the study following thyroidectomy, and angioinvasion was confirmed through histopathological examination. Venous blood (5.5 mL) was obtained and centrifuged, with serum subsequent separation and then frozen at −80 °C.

### Biochemical Measurement

The enzymatic colorimetric method on a Roche C111 device (Roche Diagnostics, Basel, Switzerland) was used to assay serum concentrations of triglycerides, low-density lipoprotein (LDL), high-density lipoprotein (HDL), cholesterol (CHOL), and C-reactive protein. Meanwhile, serum concentrations of TSH, free T3, free T4, thyroglobulin (TGB), and antithyroglobulin antibody (TGBAb) were measured using the electrochemiluminescence method on a Roche E411 device (Roche Diagnostics, Sussex, UK).

The total oxidative capacity (TOS) status was assessed using photometric immunodiagnostic assay (PerOx [TOS/TOC] kit, KC5100, 64625 Bensheim, Germany) and the total antioxidant capacity (TAC) status was determined by photometric assay (ImAnOx [TAS/TAC] Kit, KC5200, 64625 Bensheim). 8-OHdG and sortilin concentrations were evaluated using ELISA (CEA660Ge, TX 77494, USA and RAB1709 SIGMA, Sigma-Aldrich, respectively).

### Statistical Analysis

The statistical analyses were performed using GraphPad Prism 9.0 software from GraphPad Software Inc. (San Diego, CA, USA). Initially, the Shapiro-Wilk test was conducted to assess the normality of the investigated parameters. The studied parameters did not exhibit a normal distribution. As a result, nonparametric tests were used to compare the statistical differences between the groups. The data were presented as the range and median values. The Mann-Whitney test for independent groups was used to evaluate the significant differences in clinical parameters among the studied patients, with a significance level set at *P* < .05.

Furthermore, to distinguish whether the oxidative stress markers originate from the tumor itself or are a systemic phenomenon caused by the resultant peripheral processes, the logistic regression analysis was performed.

To evaluate the effectiveness of screening, receiver operating characteristic (ROC) curves were generated and the area under the ROC curves (AUC) was analyzed for distinct parameters.

Logistic regression analysis was performed to evaluate the association of combination of studied serum parameters and angioinvasion presence in PTC. AUC for the model was calculated and visualized using ROC curve. Moreover, odds ratios, screening performance characteristics such as positive predictive value and negative predictive values were obtained to describe the utility of the model.

## Results

To investigate the working hypothesis, we categorized the cohort of patients diagnosed with PTC into 2 distinct subgroups. The first subgroup consisted of individuals with PTC who demonstrated angioinvasion (referred to as the study group). The second subgroup comprised patients with PTC without these particular characteristics (referred to as the reference group). According to the guidelines provided by the 2015 American Thyroid Association, the reference group was considered to represent the very low-risk PTC group.

### Biochemical Characteristics of the Patients With PTC

In the study group, concentrations of TGB, TGBAb, CHOL, and LDL showed a significant increase, whereas HDL levels decreased compared with the reference group (*P* < .05, *P* < .05, *P* < .05, *P* < .001, and *P* < .05, respectively). However, no significant differences were observed among the groups for other parameters, as indicated in Table 2 (*P* > .05).

**Table 2.** A comparison of the biochemical profiles between the study group and the reference group

Parameter	Study group Median (minimum-maximum)	Reference Group median (minimum-maximum)	*P^a^*
TSH (µIU/mL)	0.76 (0.05-3.83)	1.38 (0.09-3.71)	.092
Free T3 (pg/mL)	2.64 (1.36-3.76)	2.41 (1.0-6.27)	.883
Free T4 (ng/mL)	1.22 (0.54-2.14)	1.83 (0.42-1.82)	.941
TGB (ng/mL)	6.41 (0.04-37.05)	1.39 (0.04-3.20)	**.041**
TGBAb (IU/mL)	11.22 (0.6-132.4)	5.99 (0.00-45.10)	**.048**
CHOL (mg/dL)	211.00 (160.00-361.00)	184.70 (99.00-262.00)	**.027**
LDL (mg/dL)	155.50 (92.00-298.00)	109.10 (60.00-175.00)	**<.001**
TG (mg/dL)	134.00 (70.00-219.00)	128.50 (84.00-151.00)	.921
HDL (mg/dL)	39.10 (29.00-56.00)	45.80 (40.00-77.00)	**.043**

Bold type indicates statistical significance.

Abbreviations: CHOL, cholesterol; HDL, high-density lipoprotein; LDL, low-density lipoprotein; PTC, papillary thyroid cancer; TG, triglyceride; TGB, thyroglobulin; TGBAb, antithyroglobulin antibodies.

^*a*^Mann-Whitney *U* test.

### Oxidative Stress Profiling Among Patients With PTC

**Figure 1.**
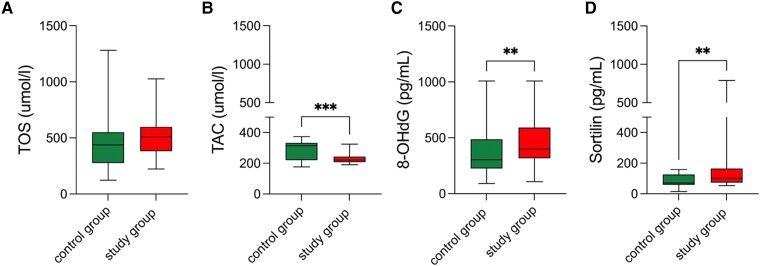
Comparison of oxidative stress marker concentrations. Abbreviations: 8-OHdG, 8-hydroxydeoxyguanosine; TAC, total antioxidant capacity; TOS, total oxidative capacity.

The analysis results revealed that the study group exhibited higher serum levels of 8-OHdG and sortilin observed compared with the reference group (*P* = .006 and *P* = .001, respectively). Conversely, the TAC concentration was lower in the study group compared with the reference group (*P* = .006) (Table 3, Fig. 1).

**Table 3.** Oxidative stress markers profiling

Parameter	Study group median (minimum-maximum)	Reference group median (minimum-maximum)	*P^a^*
TOC (µmol/L)	474.00 (223.00-1009.00)	441.00 (123.00-882.00)	NS
TAC (µmol/L)	225.00 (190.00-324.00)	326.00 (171.00-373.00)	**.0004**
8-OHdG (pg/mL)	303.42 (90.70-1008.00)	397.95 (107.60-998.00)	**.0056**
Sortilin (pg/mL)	153.5 (53.0-760)	83.62 (15.0-158.0)	**.0010**

Bold type indicates statistical significance.

Abbreviations: 8-OHdG, 8-hydroxydeoxyguanosine; NS, not significant; TAC, total antioxidant capacity; TOC, total oxidative capacity.

^*a*^Mann-Whitney *U* test.

### Logistic Regression Profiling of the Examined Parameters

Additionally, to ascertain whether the oxidative stress markers stem from the cancer itself or result from systemic processes triggered by the disease, logistic regression analysis was conducted. Our findings revealed a significant association between angioinvasion and the concentrations of TOC, TAC, sortilin, 8-OHdG, and CHOL with LDL in patients with PTC (*P* < .05) (Table 4).

**Table 4.** The angioinvasion and metastasis regression analysis

Parameter	B	SE	*P*	OR (95% CI)
The biochemical profile				
CHOL	0.014	0.814	**<.05**	1.011 (1.003-1.019)
LDL	0.025	0.250	**<.05**	1.012 (1.004-1.022)
TG	1.162	0.336	NS	1.001 (0.996-1.005)
HDL	2.844	0.721	NS	0.985 (0.960-1.010)
Glucose	1.046	0.695	NS	1.002 (0.988-1.017)
CRP	1.211	0.200	NS	1.008 (0.951-1.074)
25-OH vitamin D	1.197	0.378	NS	1.001 (0.977-1.027)
TGBAb	1.243	0.182	NS	1.002 (0.988-1.019)
TSH	1.123	0.185	NS	1.026 (0.995-1.075)
Free T3	3.148	0.688	NS	0.703 (0.412-1.132)
Free T4	6.391	0.735	NS	0.240 (0.067-0.774)
The oxidative stress markers				
TOC	2.918	0.286	**<.001**	0.997 (0.995-0.008)
TAC	7.478	0.381	**<.001**	0.996 (0.994-0.998)
Sortilin	0.528	0.603	**<.001**	1.016 (1.005-1.029)
8-OHdG	270.1	1.314	**<.001**	0.978 (0.966-0.986)

Bold type indicates statistical significance.

Abbreviations: 8-OHdG, 8-hydroxydeoxyguanosine; B, beta; CHOL, cholesterol; CRP, C-reactive protein; HDL, high-density lipoprotein; LDL, low-density lipoprotein; NS, not significant; OR, odds ratio; SE, standard error; TAC, total antioxidant capacity; TG, triglyceride; TGBAb, antithyroglobulin antibodies; TOC, total oxidative capacity.

### The Clinical Utility

**Figure 2.**
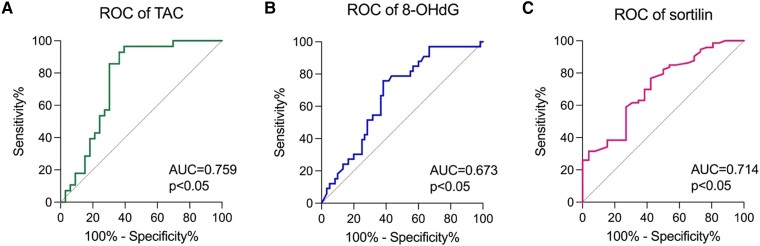
The angioinvasion screening utility of oxidative stress markers compared with AUC = 0.5. Abbreviations: 8-OHdG, 8-hydroxydeoxyguanosine; TAC, total antioxidant capacity.

To assess the significance of TAC, 8-OHdG, and sortilin in angioinvasion screening in patients with PTC, the ROC curve analysis was performed. Screening utility for TOC was not evaluated (*P* > .05). The highest screening utility was presented for TAC (AUC = 0.759), sortilin (AUC = 0.714), and 8-OHdG (AUC = 0.673) (all *P* < .05) (Fig. 2).

### Screening Panel

**Figure 3.**
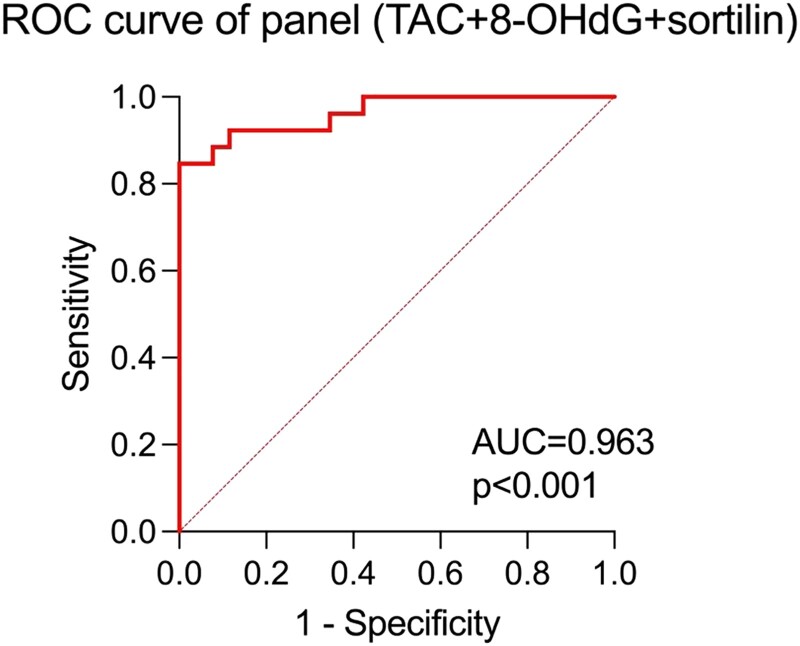
The angioinvasion screening utility of combination of markers (TAC + 8-OHdG + sortilin) compared with AUC = 0.5. Abbreviations: 8-OHdG, 8-hydroxydeoxyguanosine; TAC, total antioxidant capacity.

Logistic regression was used to evaluate the association between studied parameters and angioinvasion presence in patients with PTC. The parameters of the model and the common quality measures are summarized in Table 4 (Table 5). The AUC value obtained for a combination of TAC + 8-OHdG + sortilin (AUC = 0.963) had a higher diagnostic value than the highest AUC for a marker used separately: TAC (AUC = 0.759) (Fig. 3).

**Table 5.** Summary of the basic parameters and common quality measures of the models

Model	OR	95% CI	PPV (%)	NPV (%)	AUC	95% CI
TAC	0.9498	0.9085-0.9757	92.00	88.89	0.963	0.9173-1.000
8-OHdG	1.008	1.002-1.016				
sortilin	1.043	1.014-1.089				

Abbreviations: AUC, area under the receiver operating characteristic curve; NPV, negative predictive value; OR, odds ratio; PPV, positive predictive value.

## Discussion

### Oxidative Stress in PTC Development: Insights, Correlations, and Prospects

As the acknowledgment of oxidative stress as a substantive participant in diverse pathological cascades increases, its implications within the scope of PTC development have garnered salience. The intricate interrelationship between ROS and antioxidant defense mechanisms seems to exert a pivotal influence on the initiation and advancement of PTC (6). In our previous investigative study, we offered insights into the correlation between oxidative stress and individuals diagnosed with differentiated thyroid cancer (DTC). As a result, an elevation in the concentrations of the oxidative stress marker, particularly malondialdehyde, was observed among patients with DTC in comparison to a control group, and this elevation persisted following the administration of radioiodine (RAI) treatment (9). These findings emphasize the necessity for continued research to gain a deeper understanding of the mechanisms that underlie the increased oxidative stress among patients with DTC. Furthermore, it is imperative to investigate prospective methodologies for quantifying oxidative markers, aiming to refine the management of patients with DTC (12).

### Exploring the Link: Oxidative Status and RAI Eligibility in Patients With PTC

Thus, in the following study, we aimed to investigate the association between oxidative status and the eligibility of patients with PTC for RAI treatment. Assessing the oxidative stress status of patients could help identify those who would derive the most benefit from RAI and potentially guide personalized treatment strategies following the fact that oxidative stress markers, such as TAC, forkhead box protein O1, TOC, Sirtuin 1, tumor protein 53, and nuclear factor kappa B may serve as potential indicators for determining the eligibility and potential effectiveness of RAI therapy in managing PTC (5). This underscores a statistically significant relationship between angioinvasion and these specific biochemical markers.

### Unveiling the Potential: Oxidative Stress Markers as Screening Tools for Angioinvasion in PTC

Given the realization that oxidative stress might accelerate the progression of angioinvasion by fostering various molecular and cellular modifications that facilitate the invasive infiltration of tumor cells into blood vessels, the assessment of oxidative stress markers has garnered attention as a potential avenue to identify angioinvasion in PTC (9, 16, 17). Therefore, precise identification of angioinvasion assumes a pivotal role in determining suitable treatment approaches and predicting patient prognosis (5, 6). In the subsequent investigation, our focus was directed toward various markers associated with oxidative stress and angioinvasion, with the aim of evaluating their potential as screening tools for these conditions in the serum of patients diagnosed with PTC. This study provides evidence to support the notion that the assessment of peripheral concentrations of oxidative stress markers, specifically DNA/RNA oxidative stress damage products, Sirtuin 3, and TAC, hold promise as valuable indicators for the detection of angioinvasion and metastasis in individuals with PTC. These findings have noteworthy implications for both the prognosis and the formulation of treatment strategies for patients with PTC (18). Nevertheless, our results did not singularly identify angioinvasion, which is of paramount importance in terms of predicting PTC clinical management. Consequently, the present study aimed to evaluate the clinical utility of measuring markers of oxidative stress in identifying angioinvasion in PTC. By investigating the levels of 8-OHdG and potentially other relevant marker, such as sortilin, we sought to determine their association with angioinvasion and their potential as objective indicators of more aggressive PTC phenotype. Our study revealed that TAC, sortilin, and 8-OHdG measurement could be implemented as novel angioinvasion screening markers following their AUC = 0.76, AUC = 0.71, and AUC = 0.67 values, respectively. Additionally, our logistic regression analysis confirmed a significant association between angioinvasion in patients with PTC and the following factors, such as TOC, TAC, sortilin, and 8-OHdG, as well as the combined levels of CHOL and LDL. This highlights a statistically significant association between angioinvasion and these biochemical markers, particularly those related to oxidative stress. Nevertheless, the reviled PTC screening panel, consisting of TAC, 8-OHdG, and sortilin measurements, presented the highest clinical utility based on AUC = 0.963.

### Corroborating Insights: Comparisons With Prior Studies and Implications for PTC Management

The findings of our study are in concordance with results reported by other authors in patients with PTC, thereby concurrently validating previously established outcomes. In the study conducted by Mseddi et al, an observation of tissue overexpression of 8-OHdG was noted in patients diagnosed with PTC. This finding suggests an increased presence of 8-OHdG in the thyroid tissue of patients with PTC in their research (19). The study performed by Faulkner et al also revealed that thyroid cancer tissue is characterized by increased sortilin expression compared with benign thyroid tissues (20). In the study performed by Tabur et al, the increased serum 8-OHdG levels were also observed among patients with PTC before surgery (21). To the best of our knowledge, there have been no prior studies investigating the serum profiling of sortilin in patients with PTC. However, both sortilin and 8-OHdG measurements have been associated with more aggressive breast cancer (22, 23), pancreatic cancer (24, 25), liver cancer (26, 27), and glioblastoma (28, 29).

Our study further demonstrated a noteworthy reduction in TAC in correlation with the presence of angioinvasion. This observation suggests that in cases of angioinvasion, there is a decreased ability within the body to mitigate oxidative stress and maintain optimal antioxidant levels. Moreover, the decreased antioxidant capacity may contribute to increased oxidative damage and further promote the aggressiveness of the disease (16). The findings of this study highlight the importance of understanding the role of antioxidant systems in angioinvasion and exploring strategies to enhance antioxidant defense mechanisms to potentially mitigate the detrimental effects of oxidative stress in this context (30). These findings warrant further investigation, particularly in the context of identifying potential medical targets for intervention and treatment strategies. Furthermore, as previously proposed by Wreesmann et al, well-differentiated PTC cases that include vascular invasion but lack other aggressive characteristics do not show a notable increase in the risk of recurrence or mortality, according to existing data. Therefore, the question remains regarding the feasibility of safely managing such cases without obligatory RAI (31). Hence, our screening panel has the potential to provide additional validation as is still needed.

### Strengths and Limitations of the Study

Despite the novel angioinvasion markers measurement, our intention in measuring many biochemical parameters was to provide additional comprehensive clinical profile of patients with PTC. By including lipid profile markers (triglycerides, LDL, HDL, CHOL), we aimed to explore potential associations between lipid metabolism and PTC because dyslipidemia has been implicated in various malignancies. Additionally, the assessment of C-reactive protein allowed us to evaluate systemic inflammation, which has been linked to cancer progression. Regarding thyroid function markers (TSH, free T3, free T4, TGBAb), we aimed to assess the thyroid hormone levels and antibody presence in patients with PTC, considering the intricate relationship between thyroid function and thyroid cancer. Despite these strengths, the interpretation of the present data are limited because it constitutes a preliminary phase. Nevertheless, there are certain limitations to consider, notably the relatively low number of analyzed cases. To validate these findings on a broader scale, it is advisable to conduct a multicenter study. Unfortunately, implementing a positive control characterized by distant metastasis was unfeasible due to the scarcity of patients with PTC and distant metastasis within our institution. Additionally, our institution's preventive approach, involving routine ultrasound examinations, enables comprehensive early-stage diagnosis of PTC before the manifestation of distant metastases. Consequently, our study database includes only 5 patients with PTC distant metastases, making it insufficient for a statistically robust analysis. he primary aim of our study was to analyze markers related to angioinvasion to facilitate diagnosis and provide predictive assessments in cases of PTC (31).

## Conclusions

The recognition of angioinvasion as a prognostic factor in individuals diagnosed with PTC holds vital implications for effective management and treatment strategies. Consequently, the development of dependable screening tools that offer an objective assessment of this process assumes considerable significance. In our study, we have successfully verified the efficacy of serum oxidative stress markers as reliable indicators of angioinvasion in patients with PTC. The findings of our research suggest that the measurement of panel consisting of TAC, 8-OHdG, and sortilin could be considered an innovative screening tool for angioinvasion. Furthermore, our study revealed an association between the angioinvasion process and reduced antioxidant defense in individuals with PTC, thereby highlighting the potential for further investigation into novel therapeutic targets.

## Data Availability

The datasets analyzed during the current study are available from the corresponding author on reasonable request.

## Abbreviations

8-OHdG: 8-hydroxydeoxyguanosine
AUC: area under the receiver operating characteristic curve
CHOL: cholesterol
DTC: differentiated thyroid cancer
HDL: high-density lipoprotein
LDL: low-density lipoprotein
PTC: papillary thyroid cancer
RAI: radioiodine
ROC: receiver operator characteristic
ROS: reactive oxygen species
TAC: total antioxidant capacity
TGB: thyroglobulin
TGBAb: antithyroglobulin antibody
TOS: total oxidative capacity
